# An Ancient Street

**Published:** 1883-08

**Authors:** 


### AN ANCIENT STREET.

ONE of the oldest streets in Paris is about to disappear, as the Rue du Jour, near the Church of St. Eustache, which dates from the thirteenth century, will shortly be demolished, in connection with the improvements which are being made in this part of Paris. The Rue du Jour, so named on the *lucus a non lucendo* principle, was very narrow and gloomy, and the only building of any historic interest is an old house, now used as a china warehouse, which was known 200 years ago as the Hotel Royaumont, and was at that time the residence of Philippe Hurault, Abbot of Royaumont. At his death it became the property of François de Montmorency, who turned it to a very singular use, making it the trysting-place of all persons who were about to fight a duel. Every morning refreshments were provided in the dining-room of the Hotel Royaumont, and principals and seconds alike partook of them before proceeding to settle their differences in a room fitted up as a fencing chamber. M. de Montmorency also had a choice assortment of swords to offer those who had not weapons to their liking, and he carried his foresight so far as to keep a surgeon constantly on the premises. The duel was allowed to take place after 12 o'clock, at which hour breakfast was served for all who were in a condition to partake of it. This went on for a long time, but two women who had quarreled over their lovers fought at the Hotel Royaumont, and both were killed, and the mishap created so much stir that the Lieutenant of police forbid M. de Montmorency to accommodate any more duelists.

